# Receptor-Mediated SPION Labeling of CD4^+^ T Cells for Longitudinal MRI Tracking of Distribution Following Systemic Injection in Mouse

**DOI:** 10.3390/nano15141068

**Published:** 2025-07-10

**Authors:** Yu Ping, Songyue Han, Brock Howerton, Francesc Marti, Jake Weeks, Roberto Gedaly, Reuben Adatorwovor, Fanny Chapelin

**Affiliations:** 1 Shu Chien Gene Lay Department of Bioengineering, University of California San Diego, San Diego, CA 92093, USA; yuping@ucsd.edu (Y.P.); songyue.han@etu.utc.fr (S.H.); brhowerton@ucsd.edu (B.H.);; 2 Department of Surgery, Transplant Division, University of Kentucky, Lexington, KY 40506, USAroberto.gedaly@uky.edu (R.G.); 3 Lucille Parker Markey Cancer Center, University of Kentucky, Lexington, KY 40536, USA; 4 Alliance Research Initiative (TILT Alliance), College of Medicine, University of Kentucky, Lexington, KY 40536, USA; 5 Department of Biostatistics, University of Kentucky, Lexington, KY 40536, USA; radatorwovor@uky.edu; 6 Department of Radiology, University of California San Diego, San Diego, CA 92093, USA

**Keywords:** CD4^+^ T cells, SPION, MRI, magnetic-activated cell sorting, immunomodulation, transplantation

## Abstract

Tracking T cells in vivo using MRI is a major challenge due to the difficulty of labeling these non-phagocytic cells with a sufficient contrast agent to generate a detectable signal change. In this study, we explored CD4-Superparamagnetic iron oxide nanoparticles (SPION), which is commonly used in magnetic cell sorting, as a potential receptor-mediated, specific CD4^+^ T cell MRI labeling agent. We optimized the labeling protocol for maximal CD4^+^ cell labeling and viability. Cell health was confirmed with trypan blue assay, and labeling efficacy was confirmed with Prussian blue staining, transmission electron microscopy, and MRI of labeled cell pellets. Key cell functionality was assessed by flow cytometry. Next, CD4-SPION-labeled T cells or unlabeled T cells were delivered via intravenous injection in naïve mice. Liver MRIs pre-, 24 h, and 72 h post-T cell injection were performed to determine in vivo tracking ability. Our results show that CD4-SPION induces significant attenuation of T2 signals in a concentration-dependent manner, confirming their potential as an effective MRI contrast agent. In vitro, analyses showed that CD4^+^ T cells were able to uptake CD4-SPION without affecting cellular activity and key functions, as evidenced by Prussian blue staining and flow cytometric analysis of IL-2 receptor and the IL-7 receptor α-chains, CD69 upregulation, and IFN-γ secretion. In vivo, systemically distributed CD4-SPION-labeled T cells could be tracked in the liver at 24 and 72 h after injection, contrary to controls. Histological staining of tissue sections validated the findings. Our results showed that SPION CD4^+^ T cell sorting coupled with longitudinal MR imaging is a valid method to track CD4^+^ T cells in vivo. This safe, specific, and sensitive approach will facilitate the use of SPION as an MRI contrast agent in clinical practice, allowing for non-invasive tracking of adoptive cell therapies in multiple disease conditions.

## 1. Introduction

Harnessing one’s own immune machinery, in particular, using T cells in therapeutic interventions, has greatly changed the treatment landscape of various malignant tumors [1] and immune-related diseases, including diabetes [2] and allograft rejection in transplantation [3]. The use of T cells as a cancer-killing agent was first developed by Rosenberg et al. [4] and has since seen unprecedented development in the oncology field [5,6]. Through numerous rounds of genetic engineering innovations, chimeric antigen receptor (CAR) T cell therapies [1,7,8] have gone from lab-to-bedside success and continue to inspire researchers in treating other diseases through these strategies. Branching out from cytotoxic (CD8^+^) T cells for cancer therapy, helper T cells (CD4^+^) have attracted considerable attention because of their role in the regulation of immune responses [7,8]. Specifically, regulatory T cells (Tregs), a subset of helper cells, have emerged as potential therapy candidates in a variety of autoimmune, allergic, and transplantation-associated diseases [9,10,11]. These diseases are associated with failure of immune self-tolerance, in which restoration of immune homeostasis is a therapeutic priority [12,13]. Clinically used immunosuppressive drugs are overall effective, but come with potential side effects including cardiotoxicity [14], nephrotoxicity, and diabetes [15]. For this reason, Treg and CAR Treg therapies are being investigated to improve disease outcomes or graft tolerance. However, the success of Treg transfer is closely related to the ability to accurately track the biodistribution and persistence of the administered cells, which is crucial for assessing the therapeutic efficacy and optimizing the treatment regimen [16,17].

Currently, biopsy followed by histological staining or flow cytometry represents the gold standard for the determination of in vivo cellular biodistribution [18,19]. However, these approaches are time-consuming, tissue-destructive, and prone to sampling errors [20,21,22]. Recent work on label-free optical biopsies can retrieve spatial and molecular information, but are only non-destructive to the sample specimen, and still require invasive collection [23]. Magnetic resonance imaging (MRI) is a non-invasive imaging technique with high spatial resolution and excellent soft-tissue contrast, and has been widely used in clinical practice [24,25,26,27,28,29]. The integration of contrast agents has further enhanced the capabilities of MRI, especially in the field of in vivo cell tracking [28,29,30,31]. Specifically, superparamagnetic iron oxide nanoparticles (SPION) are good candidates for labeling and tracking of cells, due to their low cytotoxicity [32] and advantageous MR properties to reduce the T2 relaxation time of neighboring protons [25,30,33,34,35,36,37]. However, there are significant challenges in labeling non-phagocytic cells (e.g., helper T cells, Tregs), mainly due to their limited phagocytic and cytoplasmic capacity, in addition to concerns for cell viability and function [38,39].

To date, antibody-mediated labeling of lymphocytes with micron-sized iron oxide particles (MPIO) has only been confirmed to provide MRI contrast in vitro [40]. For T cells, only cell-penetrating peptide-ornated contrast agents [41,42] or targeted SPION such as CD25-SPION [35] have successfully labeled T cells with detectable signal change on MRI [24,43,44]. Other agents, including very small superparamagnetic iron oxide particles (VSOP) for central nervous system inflammation and micron-sized iron oxide nanoparticles (MIONPs) for post-transfer accumulation, likewise produce hypointense signals on T2-weighted images (Table A1). However, these approaches remain limited by non-specific iron retention and suboptimal sensitivity for sparse or early-stage infiltrates (Table A1). A helper T cell contrast agent that combines safety, specificity, clinical translatability, and the possibility of longitudinal studies remains to be found.

Of interest for specificity and clinical translation is the recent study that explored the dual use of SPION specifically designed for the magnetic separation of CD25^+^ T cells from peripheral blood mononuclear cells (PBMC) as an MRI contrast agent [35,45]. Cell sorting microbeads are designed to specifically bind to a given receptor on the surface of target cells, in this case, T cells [35,46,47]. A heterogeneous cell suspension is then placed in a magnet, and cells bound to the magnetic SPION bead are captured by the magnet while unlabeled cells are washed away [35,46]. This method yields very pure cell populations in the upwards of 90%. The advantage of this targeting approach is that it ensures specific labeling of the cell population of interest and, therefore, specific labeling for MRI applications.

Here, we are interested in labeling helper cells through the CD4 receptor. When CD4-SPION binds to its target receptors on T cells, it initiates receptor-mediated endocytosis [48,49]. During this process, the cell membrane invaginates to envelop the SPION-receptor complexes, forming endocytic vesicles [48,49]. These vesicles then transport the SPION inside T cells [50], encapsulating them with endosomes and lysosomes and thereby enabling long-term retention of the contrast for MRI applications [35,42]. In this study, we optimized the labeling procedure to maximize internalization of the CD4-SPION and explored the feasibility of in vivo longitudinal CD4^+^ T cell tracking in liver tissue following systemic injection.

## 2. Materials and Methods

### 2.1. Contrast Agent: CD4-SPION

Magnetically-assisted cell sorting (MACS) CD4 microbeads (Clini-MACS, Miltenyi Biotec, Somerville, MA, USA) were used to selectively enrich T cells and label them. These SPIONs consist of CD4 antibodies attached to a dextran-coated iron core with an average diameter of approximately 50 nm [38] and a low polydispersity index (<0.2). Iron concentration was determined using an Agilent 7900 Inductively Coupled Plasma Mass Spectrometer (ICP-MS)(Agilent, Santa Clara, CA, USA) at the University of Kentucky Center for Applied Energy Research. The instrument was calibrated using a 5-point serial dilution of a 100 ppm Iron standard purchased from VHG-LGC. Tellurium was used as the internal standard. Samples were digested for 72 h in 5 vol% trace metal grade concentrated nitric acid. Appropriate serial dilutions (1000, 100, 10×) were made and introduced to the instrument. CD4-SPION samples were found to have an iron concentration of 0.116 mg Fe/mL.

### 2.2. Human CD4^+^ T Cell Isolation and Labeling

The study was approved by the University’s Institutional Review Board (IRB #48583). Peripheral blood mononuclear cells (PBMC) were isolated from the plasma of an anonymous donor (Kentucky Blood Center, Lexington, KY, USA or San Diego Blood Bank, San Diego, CA, USA) using leukapheresis. PBMC were processed by density gradient centrifugation using Lymphoprep (STEMCELL, Cambridge, MA, USA). T cells were purified from PBMC using the EasySep Human T Cell Isolation Kit (STEMCELL, Cambridge, MA, USA) and then further purified using CD4 microbeads (positive sorting) or CD8 microbeads (Negative sorting, Miltenyi Biotec, Waltham, MA, USA) to enrich CD4^+^ T cells. Both positive and negative sorting yielded a pure CD4 cell population (CD4^+^ > 90%, determined by flow cytometry), and negative sorting was chosen to prevent ‘contamination’ with CD4-SPION of the negative control. These CD4^+^ T cells were cultured in RPMI 1640 medium (Corning, Corning, NY, USA) with 10% fetal bovine serum (FBS, Corning, Corning, NY, USA) and 1% penicillin-streptomycin (PS, VWR, Radnor, PA, USA). Cells were activated after isolation (day 1) and then reactivated on day 14 using T cell TransAct per manufacturer’s instructions (Miltenyi Biotec, Waltham, MA, USA). Recombinant human IL-2 (Miltenyi Biotec) at a concentration of 100 units/mL was supplemented every other day. Cells were cultured at 37 °C, 5% CO_2_ for 14 days to achieve the desired cell numbers.

### 2.3. CD4^+^ T Cell Labeling and Prussian Blue Staining

For cell labeling, negatively selected CD4^+^ T cells were plated in 24-well plates at a density of 1 million cells in 0.5 mL of RPMI, supplemented with 10% FBS, 1% PS, and 100 units/mL recombinant human IL-2. Cells were incubated for 24 h with various amounts of CD4-SPION (2.5, 5, 10, or 20 μL per sample). Unlabeled CD4^+^ T cells were plated in matching conditions and served as controls. Labeled CD4^+^ T cells or unlabeled control cells were washed twice with 5 mL Dulbecco’s Phosphate-Buffered Saline (DPBS) (Corning, Corning, NY, USA) to remove excess label. Cells were resuspended in 100 μL DPBS and spread onto histology slides (Superfrost slide, Fisherbrand, Waltham, MA, USA). Slides were allowed to air-dry before cells were fixed with 10% Formalin (VWR, Radnor, PA, USA) for 15 min. Iron staining was performed with a Prussian blue staining kit (Polysciences, Warrington, PA, USA) consisting of a 1:1 mixture of Hydrochloric acid (HCl) and Potassium Ferrocyanide for 15 min, and cytosol staining was subsequently performed with Nuclear Fast Red (NFR) made of a 1:1 mixture of NFR and DPBS for 1 min. Permount (Fisher Scientific, Waltham, PA) was used to mount the coverslip. Photos were acquired on a Nikon Ti-U Microscope (Nikon, Tokyo, Japan) with a 60× objective.

### 2.4. Electron Microscopy of CD4-SPION-Labeled T Cells

SPION-labeled and unlabeled control CD4^+^ T cells were fixed in 4% paraformaldehyde and 3.5% glutaraldehyde at 4 °C for 1 h followed by 3 washes in sucrose for 5 min. The cells were dipped in 1% OsO_4_ at 4 °C for 1 h. Cells were then resuspended in 0.1 M buffer and dehydrated at 4 °C in an ascending series of ethanol baths and finally 2× propylene oxide at room temperature. The cells were infiltrated overnight in a 1:1 mixture of PO and epoxy resin. The next day, the cells were resuspended in fresh resin and polymerized for 48 h at 60 °C. Selected areas were thin sectioned on a microtome at 60–80 nm, mounted on copper 300 mesh grids and then stained with uranyl acetate and lead citrate. Grids were examined using a FEI Talos F200X transmission electron microscope(Thermo Fisher Scientific, Waltham, MA, USA) and STEM and HAADF images and EDS spectra were acquired using inbuilt software and digital camera.

### 2.5. ICP-OES Measurement of Cellular Iron Uptake

Triplicate 1 million T cells were seeded in 48-well plates and incubated with various SPIONs at optimal dosage (40 μL per ml for CD4 and CD25 SPIONs and 400 μg Fe/mL for Synomag) in 500 μL complete culture medium. After overnight incubation, cells were washed thoroughly three times with phosphate-buffered saline (PBS) to remove excess contrast agent. Dry cell pellets were digested in 1 ml of concentrated nitric acid (65%) and 200 μL hydrogen peroxide (30%) and incubated at 90 °C for 1 h. Samples were then diluted to a final volume of 10 mL with ultrapure deionized water. Iron content was quantified using an ICP-OES system (Thermo Scientific, Waltham, MA, USA). Calibration standards were prepared from a certified iron standard solution and diluted to generate a standard curve covering the expected sample range (0.1–1000 ppb Fe in dH*_2_*O). Total iron uptake was normalized to cell number (determined by manual count prior to digestion). Data were reported as picogram of iron per cell.

### 2.6. MRI Measurement of CD4-SPION-Labeled T Cell Phantoms

One million labeled (per protocol above) or control cells were washed and cell viability was assessed using Trypan Blue assay (Corning, Corning, NY, USA). Cells were then washed in DPBS and resuspended in 100 µL of 1.5% agarose (Sigma Aldrich, St. Louis, MO, USA) solution in PCR tubes. The MRI phantoms were scanned using a 7T MRI system (Bruker, Billerica, MA, USA) using a commercial mouse birdcage body coil (Bruker ^1^H mouse body coil with 40 mm inner diameter). Axial images of the cell samples were acquired using a Turbo RARE sequence with parameters TR/TE = 2500/33 ms, Matrix size = 128 × 128, FOV = 28 × 28 mm^2^, Slice thickness = 0.7 mm, Averages = 2, Slice number = 16 and a T2map Multi Slice Multi Echo (MSME) sequence with TR = 2000 ms and 14 TEs equally spaced from 7 to 90 ms, Matrix size = 96 × 96, FOV = 28 × 28 mm^2^, Slice thickness = 0.7 mm, Averages = 1, Slice number = 16. T2 values were extracted using the system’s inbuilt Image Sequence Analysis (ISA T2vtr) software by defining circular ROIs over each tube and averaged over 3 slices. T2 maps were saved using the same software, and a custom color lookup table was created to aid visualization of the T2 values.

### 2.7. Functional Test of CD4-SPION-Labeled T Cell

To assess early activation, triplicate SPION-labeled and unlabeled CD4^+^ T cells (1 × 10^6^) were stimulated with TransAct T cell activation reagent (Miltenyi Biotec; 20 µL/mL) for 18–24 h at 37 °C, 5% CO_2_. Cells were collected, washed once in cold FACS buffer (PBS + 2% FBS), and stained for viability with Zombie Aqua™ Fixable Viability Kit (BioLegend, San Diego, CA, USA; 1:1000 in PBS for 15 min at RT). After washing, cells were stained with 0.5 µL antibody cocktail in 100 µL FACS buffer for 15 min on ice in the dark using fluorochrome-conjugated antibodies: anti-CD69 (FITC; BioLegend #310916), anti-CD25 (PerCP/Cy5.5; BioLegend #356112), and anti-CD127 (PE/Cy7; Invitrogen #25-1278-42). Cells were washed twice and acquired within 3 h.

For intracellular staining to measure IFNγ production, triplicate SPION-labeled and unlabeled CD4^+^ T cells were stimulated with TransAct (20 µL/mL) for 40–48 h at 37 °C, 5% CO_2_. Four hours prior to harvest, Cell Activation Cocktail with Brefeldin A (BioLegend #423303; 50 µL/well) was added to trap cytokines intracellularly. Cells were washed, stained for viability (Zombie Aqua™, as above). Fixation and permeabilization were performed using the FoxP3 Fix/Perm Buffer Set (BioLegend) for 40 min at RT. After two washes in permeabilization buffer, cells were stained for 30 min at RT with anti-IFNγ (BV421; BioLegend #502532) in permeabilization buffer. Cells were washed twice, resuspended in FACS buffer, and acquired within 2 h.

All data were acquired on a BD LSR Fortessa X-20 flow cytometer (BD Biosciences, Franklin Lakes, NJ, USA) collecting ≥50,000 events per sample. Data were analyzed using FlowJo v10 (Tree Star, Ashland, OR, USA). Cell viability was determined by exclusion of Zombie Aqua™–positive events. Surface and intracellular marker expression were quantified as percentage positive as indicated.

### 2.8. In Vivo MRI Tracking of CD4-SPION-Labeled T Cells

Animal experiments were approved by University of Kentucky’s institutional animal care and use committee (IACUC #2019-3341) and all experiments were conducted in accordance with IACUC guidelines and reported in accordance with ARRIVE guidelines. Fresh human helper cells (CD4^+^) cells were incubated for 16 h with 10 μL CD4-SPION per million cells (2.32 μg Fe/mL) and maintained at a concentration of 2 × 10^6^ cells/mL of full media in 6-well plates. After incubation, excess SPION were removed by three washes followed by centrifugation. Unlabeled fresh CD4^+^ T cells were prepared under the same conditions. Cell samples were resuspended at a concentration of 2 × 10^7^ cells in 200 μL PBS for intravenous injection. Fourteen female NSG mice were sourced from Charles River. Liver MRI prescans were acquired for all mice prior to intravenous cell injections. Seven mice received CD4-SPION-labeled T cells, seven mice received unlabeled CD4^+^ T cells (negative control). Liver MRIs were acquired 24 h and 72 h post-injection.

MRI images were acquired on the same system and coil as above (Bruker BioSpec 7T, Billerica, MA, USA). Axial images of mouse liver were acquired using a Turbo RARE sequence with parameters TR/TE = 2000/20 ms, Matrix size = 128 × 128, FOV = 36 × 24 mm^2^, Slice thickness = 1.2 mm, Averages = 2, Slice number = 16 and a T2map Multi Slice Multi Echo (MSME) sequence with TR = 2000 ms and 10 TEs equally spaced from 6.4 to 64 ms, Matrix size = 128 × 128, FOV = 36 × 24 mm^2^, Slice thickness = 1.2 mm, Averages = 3, Slice number = 16. T2 values were extracted using the system’s inbuilt Image Sequence Analysis (ISA T2vtr, Paravision 360, V3.0) software by defining three circular liver ROIs over 3 slices (9 measurements per mouse per time point). ROIs were drawn by a blinded, experienced radiologist across different liver Couinaud segments, avoiding large vessels such as the hepatic and portal venous branches. T2 maps were saved using the same software, and a custom color lookup table was created to aid visualization of the T2 values.

### 2.9. Histological Analyses

Immuno-histo-chemistry: After the last imaging timepoint, animals were euthanized via CO_2_ inhalation and liver tissues were harvested and fixed in 10% formalin for 24 h. Livers were then dehydrated through a series of increasing concentrations of ethanol (70%, 90%, 100%) and xylene. Human spleen samples were used as positive control and naïve mouse livers were used as negative control. Tissues were then embedded in paraffin and cut to 5 μm slices using a microtome. Tissue sections were baked at 60 °C for 1 h and rehydrated with xylene and ethanol (100%, 95%, 70%) before immersion in distilled water. Antigen dissociation was carried out using an antigen unmasking solution (Vector Laboratories, Newark, CA, USA) at 95 °C for 30 min. Peroxidase activity was blocked with Bloxall (Vector Laboratories, Newark, CA, USA) for 10 min, followed by protein blocking with Blotto (ThermoFisher Scientific, Waltham, PA, USA) for 10 min. Anti-human CD3 primary antibody (rabbit host, Abcam, Cambridge, UK) was applied to the tissue section and kept at room temperature for 1 h. After washing, anti-rabbit HRP polymer (Cell IDx, San Diego, CA, USA) was applied for 30 min, followed by the addition of DAB (VWR, Radnor, PA, USA) for 5 min to produce a brown precipitate. Finally, the sections were counterstained with Nuclear Fast Red (Abcam, Cambridge, UK) for 5 min and dehydrated in 95% alcohol followed by absolute alcohol. Slides were mounted with Cytoseal 60 (Fisher Scientific, Waltham, PA, USA).

Prussian blue staining: The sections were stained with Prussian blue to detect iron-labeled cells. Prussian blue working solution was made by mixing equal amounts of potassium ferricyanide and hydrochloric acid. The sections were incubated in this solution twice for 10 min each. After rinsing with distilled water, the sections were stained with nuclear fast red for 2 min. Then, the sections were rapidly dehydrated with ethanol and cleared with Citrisolv (Decon Laboratories, King of Prussia, PA, USA) and finally sealed with Cytoseal 60.

Slide imaging was performed with Aperio AT2—Digital Whole Slide Scanner (Leica, Wetzlar, Germany, Version 102.0.4.6) and analyzed with ImageScope (Aperio) software (Leica, Version 12.4.0.5043).

### 2.10. Statistical Analyses

We performed descriptive summaries for all the data by reporting the mean and standard deviation for the continuous outcome for each group (CD4-labeled T cell vs Unlabeled T cell mice. A T-test was utilized to compare the outcome from the two groups at each time point. A change from baseline outcome was calculated as the post-baseline outcome minus the baseline outcome value. The change from the baseline outcome removes any baseline differences in measurement that may affect the post-baseline outcome values (e.g., ΔT2_24h_ = (T2_pre_ − T2_24h_), ΔT2_72h_ = (T2_pre_ − T2_72h_)).

A one-way ANOVA model was fitted to analyze the change from baseline outcome by comparing the two groups at each time point. The results were consistent with those obtained from the two-sample *t*-test that was conducted. A non-parametric model, specifically the Wilcoxon test, was used to evaluate the fit of the above model and determine if the results will differ due to the small sample size. The findings from the non-parametric tests aligned with the earlier results. The standard 5% significance level was used for all hypothesis testing. All statistical analyses were performed using SAS software Version 9.4 (TS1M1, SAS Institute, Cary, NC, USA).

## 3. Results

### 3.1. Confirmation of CD4-SPION Internalization by CD4^+^ T Cells

**Figure 1:**
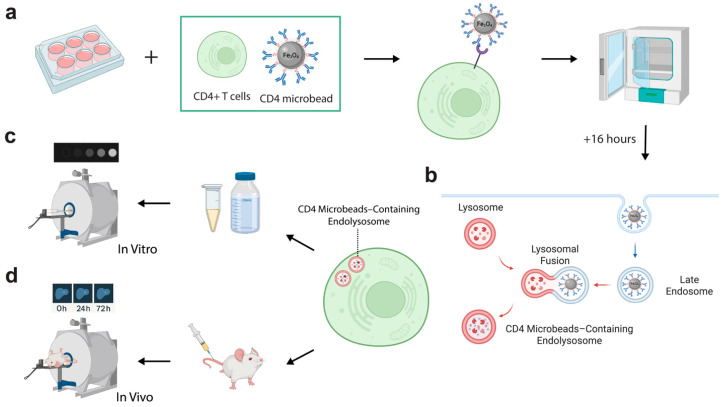
Schematic of CD4-SPION receptor-mediated labeling of CD4^+^ T cells and subsequent in vitro and in vivo MRI. (**a**) CD4^+^ T cells isolated from human peripheral blood mononuclear cells are incubated with antibody–coated CD4-SPION in culture media at 37 °C for 16 h to promote receptor-mediated internalization. (**b**) Representation of the intracellular fate of CD4-SPION. CD4-SPION first triggers endocytosis via the CD4 antibody and later fuses with endolysosomal compartments. (**c**) Representative in vitro T_2_-weighted images of cell pellets. SPION-labeled and unlabeled T cells are embedded in agarose phantoms and scanned on a 7 T MRI system to assess T_2_-weighted signal attenuation. (**d**) Representative in vivo liver MRI. Labeled or unlabeled T cells are infused intravenously into mice; liver images are acquired at baseline (0 h) and at 24 h and 72 h post-infusion.

**Figure 2:**
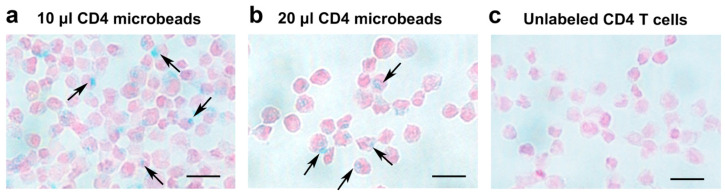
Histological Confirmation of CD4-SPION uptake by CD4^+^ T cells using iron staining. Shown are light microscopy images of CD4^+^ T cells labeled with different concentrations of CD4-SPION. CD4^+^ T cells labeled overnight with 10 μL (**a**) or 20 μL (**b**) CD4-SPION exhibit positive Prussian blue staining (**a**,**b**) compared to non-labeled CD4^+^ T cells (**c**). Scale bar represents 20 μm. The black arrows indicates CD4-SPION internalization.

**Figure A1:**
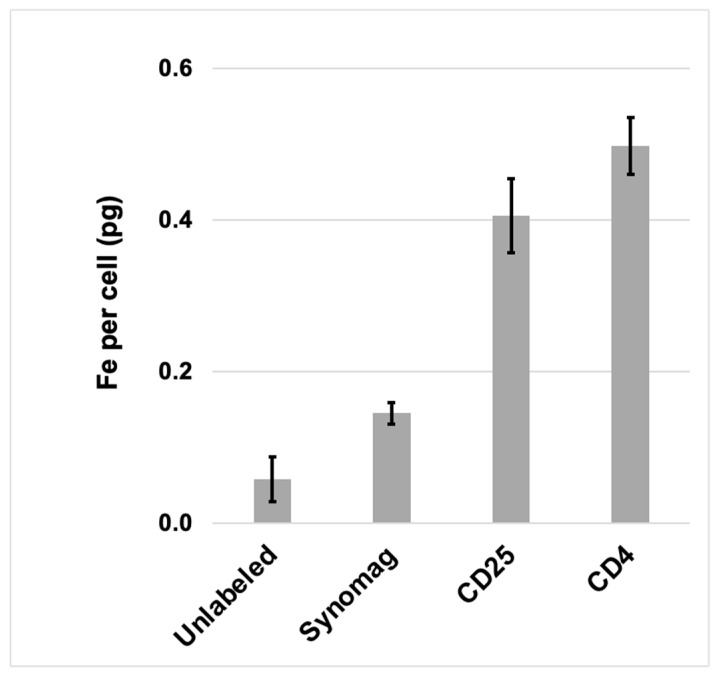
Comparison of various SPION uptake by CD4^+^ T cells. ICP-OES quantification shows iron uptake per cell for CD4-SPION (0.50 ± 0.037 pg Fe/cell) compared to CD25-SPION (0.41 ± 0.049 pg Fe/cell, NS), Synomag (0.15 ± 0.014 pg Fe/cell, *p* < 0.05) and unlabeled controls (0.06 ± 0.029 pg Fe/cell, *p* < 0.05). Mean ± SD values are shown.

CD4^+^ T cell labeling with CD4-SPION was optimized by varying incubation time and contrast agent concentration (Figure 1a). In all test conditions, T cell viability remained unaltered (>92% viable cells, *p* < 0.010 compared to unlabeled T cells). Prussian blue staining revealed successful intracellular localization of the contrast agent, indicating that the cells had internalized the SPION (Figure 2a,b). Unlabeled control cells showed no positive staining (Figure 2c). ICP-OES experiments confirmed that CD4^+^ T cells could be effectively labeled with CD4-SPIONs (0.50 ± 0.037 pg Fe/cell), as compared to labeling with CD25-SPION (0.41 ± 0.049 pg Fe/cell, NS) and Synomag (0.15 ± 0.014 pg Fe/cell, *p* < 0.05, commercial contrast agent) (Figure A1). These results demonstrate the potential of using CD4-SPION as a cell tracking agent.

**Figure 3:**
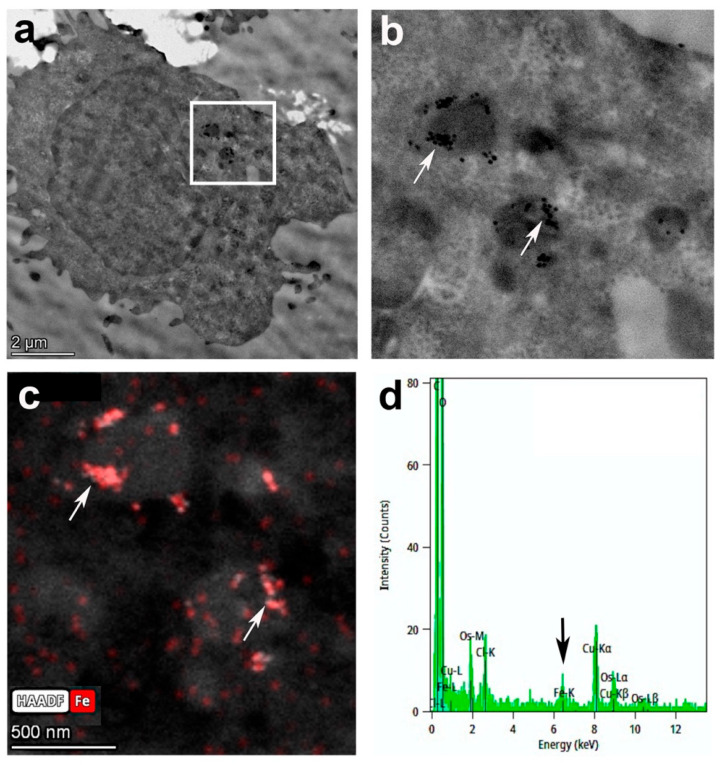
Transmission electron microscopy of labeled CD4^+^ T cells. Full cell image (**a**) and corresponding window of magnified inset (**b**) show a punctate distribution of iron SPION (arrows) in cell lysosomes or endosomes. Iron-specific filter (red) on STEM mode (**c**) confirms SPION localization, and the Fe-K peak (arrow) on the Energy Dispersive Spectroscopy (EDS) spectrum corroborates the presence of large amounts of iron in area imaged (**d**).

Transmission electron microscopy (TEM) images clearly demonstrated that the CD4-SPION not only adhered to the cell surface but were internalized within intracellular compartments (Figure 1b), primarily endosomes and lysosomes (Figure 3a,b). To further confirm the precise localization of the iron-containing SPION, we utilized scanning transmission electron microscopy (STEM) with an iron-specific filter (displayed in red, Figure 3c). Punctate circular SPION could be detected in these compartments (Figure 3b,c). Elemental analysis was performed using Energy Dispersive X-ray Spectroscopy (EDS). The resulting spectrum revealed a Fe-K peak, showing the presence of substantial iron content within the cells (Figure 3d), corroborating the successful internalization and retention of the iron-oxide core of the CD4-SPION. Unlabeled control cells did not present Fe-K EDS peaks.

### 3.2. Evaluation of In Vitro MRI Signal Intensity Effect of CD4-SPION-Labeled T Cells

**Figure 4:**
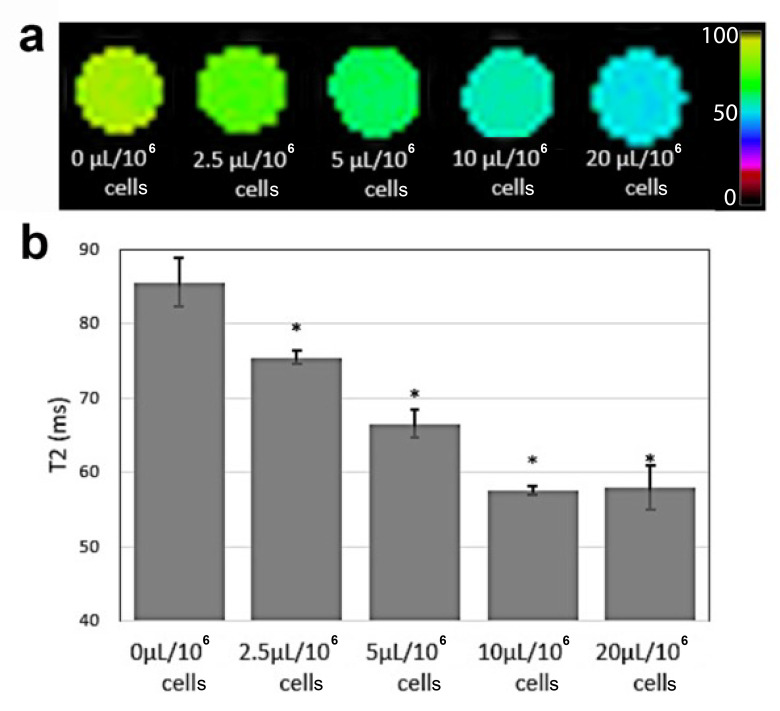
MRI of labeled CD4^+^ T cell phantoms. Color-coded T2-weighted magnetic resonance image (**a**) and corresponding T2 values (**b**) of agarose phantoms containing CD4^+^ T cells labeled overnight with varying levels of CD4-SPION (2.5–20 μL per million T cells) shows significant MRI signal reduction compared to unlabeled CD4^+^ T cells (* *p* < 0.05).

Labeled and unlabeled CD4^+^ T cells were embedded in agarose phantoms for in vitro MRI to measure T2 relaxation changes (Figure 1c). CD4-SPION-labeled T cells exhibited a concentration-dependent decrease in signal intensity on T2-weighted MR images compared to unlabeled cells (Figure 4a). This signal attenuation is consistent with the presence of superparamagnetic species (CD4-SPION) inside the T cells. Quantitative analysis revealed that T cells labeled with CD4-SPION at any of the tested concentrations yielded significant T2 decrease (*p* < 0.050) compared to unlabeled cells (T2 = 85.6 ± 4.02 ms, Figure 4b). Concentrations of 10 μL and 20 μL per million T cells demonstrated maximal reduction in T2 signal intensity (T2 = 57.6 ± 0.74 ms and T2 = 57.9 ± 3.67 ms, respectively) and T cell uptake seemed to reach a plateau possibly due to the maximum load of SPION into the cell (Figure 4b). Specifically, both concentrations resulted in a ~30% decrease in T2 signal compared to unlabeled control cells (*p* < 0.005), which motivated in vivo tracking feasibility studies.

### 3.3. Assessment of Phenotype, Activation Markers, and Cytokine Production in CD4-SPION-Labeled T Cells

**Figure 5:**
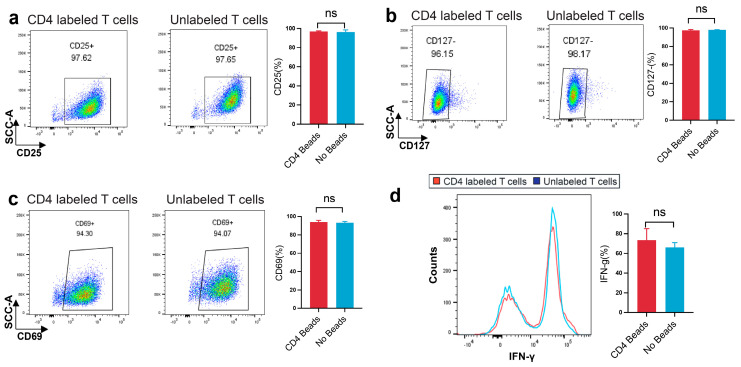
CD4-SPION labeling preserves CD4^+^ T cells phenotype, early activation expression, and cytokine production. (**a**) Dot plots of CD25 expression versus side scatter (SSC-A) on CD4-SPION-labeled and unlabeled T cells; the gated CD25^+^ population defines α-chain of the IL-2 receptor (percentage inset). Bar plot summarizes mean ± SEM Treg frequency for labeled versus unlabeled cells. (**b**) Dot plots of CD127 expression under the same activation conditions reveal equivalent proportions of CD127^+^ on CD4-SPION-labeled and unlabeled T cells; the gated CD127^−^ population defines the α-chain of the IL-7Rα. Bar plot summarizes mean ± SEM Treg frequency for labeled versus unlabeled cells. (**c**) Dot plots of CD69 expression for labeled and unlabeled T cells; percentage of CD69^+^ cells is indicated. Bar graph shows mean ± SEM CD69^+^ frequency for both conditions. (**d**) Histogram overlay of IFN-γ staining in CD4-SPION-labeled (red) and unlabeled (blue) cells. Bar graph displays mean ± SEM percentage of IFN-γ^+^ cells. Paired t-test was used for all comparisons; (n = 3); ns = not significant, *p* > 0.05.

T cell activation and effector function critically depend on CD4 co-receptor engagement to initiate signaling cascades that drive early activation marker upregulation and cytokine secretion [11,51]. Therefore, we compared CD25^+^ and CD127^−^ expression, as well as the early activation marker CD69, and intracellular IFN-γ production between CD4-SPION-labeled and unlabeled CD4^+^ T cells [51]. CD25 and CD127 subset frequency is unchanged by CD4-SPION labeling (Figure 5a,b). Flow cytometric analysis of CD25 and CD127 expression demonstrated that the proportion of CD25^+^CD127^−^ was comparable between CD4-SPION-labeled (97.6 ± 0.8%, 96.2 ± 0.6%) and unlabeled (97.7 ± 0.5%, 96.2 ± 0.7%) cells (n = 3, *p* = 0.6568; n = 3, *p* = 0.3324 by paired *t*-test). These data indicate that CD4-SPION binding does not alter the surface expression of the IL-2 receptor α-chain (CD25) or the IL-7 receptor α-chain (CD127). Assessment of side scatter (SSC-A) versus CD69 expression revealed similar expression of CD69^+^ cells following 18–24 h stimulation in both groups (94.30 ± 1.5% for labeled vs. 94.07 ± 1.3% for unlabeled; n = 3; *p* = 0.599, indicating that early activation marker CD69 is not affected by labeling (Figure 5b). Histogram overlays of IFN-γ staining and corresponding quantification showed no significant difference in the percentage of IFN-γ^+^ cells between labeled (75.8 ± 4.6%) and control (68.9 ± 5.2%) T cells (n = 3; *p* = 0.37; Figure 5c). Thus, cytokine-producing capacity is fully retained following CD4-SPION labeling. Collectively, these phenotypic results demonstrate that CD4-SPION labeling preserves key functionality of CD4^+^ T cell phenotype, early activation, and effector cytokine production, supporting its suitability for MRI-based tracking without compromising T cell activity.

### 3.4. In Vivo MRI of CD4-SPION-Labeled T Cells in Mouse Liver 24 h and 72 h Post-Injection

**Figure 6:**
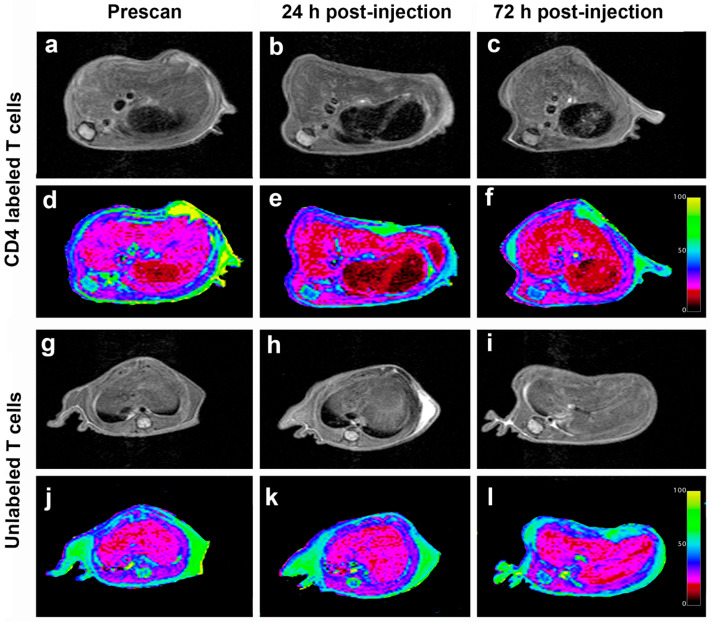
Longitudinal in vivo MR images of mice receiving intravenous injection of CD4-SPION-labeled T cells. Representative axial liver images of CD4-SPION-labeled T cell receiving mouse pre (**a**), 24 h (**b**), and 72 h (**c**) -post IV injection show T2 signal reduction, as assessed via T2 mapping ((**d**–**f**), respectively, dark red signal replacing magenta baseline) compared to unlabeled T cells (**g**–**l**).

**Figure 7:**
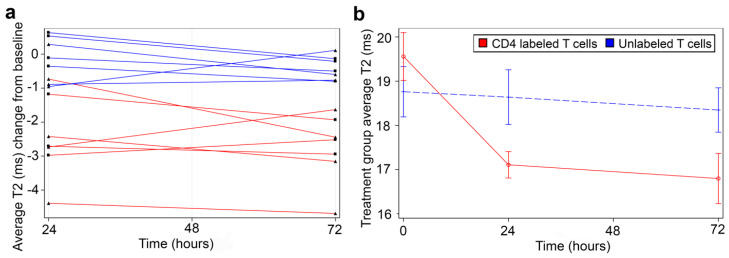
Quantitative summary of in vivo T2 measurements. (**a**) Mice receiving CD4-SPION-labeled T cells 24 h and 72 h post-injection had significantly shorter T2 relaxation times compared to the pre-scan value (red datapoints). In contrast, mice receiving unlabeled T cells showed no significant change in T2 (blue datapoints). Data are expressed as mean T2 average change from baseline per animal, with nine measurements per data point per animal. Each triangle and square means a single individual. (**b**) The difference in T2 between mice receiving CD4 T cells and mice receiving CD4-SPION-labeled T cells was also statistically significant at 24 h and 72 h post-infusion (*p* = 0.0014 and *p* = 0.0005, respectively). T2 change of CD4-SPION-labeled T cells between 24 h and 72 h post-infusion time point shows no significance (*p* = 0.6372). Data are expressed as mean T2 average of seven mice per group and nine measurements per data point per animal.

Mouse abdomens were imaged prior to T cell infusion and 24 h and 72 h post-infusion (Figure 1d). Mice injected with CD4-SPION-labeled T cells exhibited visibly shortened liver T2 relaxation times compared to the control group at both post-infusion (Figure 6), as evidenced by the liver area changing from magenta to diffuse dark red on T2 maps (Figure 6d–f). MRI analysis revealed significant differences in T2 relaxation times between mice receiving CD4-SPION-labeled T cells and those receiving unlabeled T cells. Specifically, individual mice had a ΔT2_24h_ (T2_pre_ − T2_24h_) of 2.4 ± 1.2 ms compared to mice receiving unlabeled controls (ΔT2_24h_ = 0.1 ± 0.6 ms, Figure 7a) with a significant difference (*p* = 0.001) between the average decrease in relaxation time. This reduction in CD4 T cell counts corresponds to a 12.5% decrease, while the decrease in the unlabeled T cells represents only a 0.7% reduction. By 72 h, the significant difference became even more pronounced, with an average decrease of 2.8 ± 1.0 in the relaxation time for the mice that received the CD4 T labeled cells compared to an average decrease of 0.4 ± 0.3 in the unlabeled T cell mice. T2 signal decay in the labeled cell recipients remained constant until the day three timepoint (ΔT2_72h_ = −2.34 ± 0.7 ms) indicating sustained detectability of the labeled cells. T2 relaxation times between mice receiving unlabeled T cells and those receiving CD4-SPION-labeled T cells were significantly different at both 24 h and 72 h post-infusion (*p* = 0.001 and 0.001, respectively, Figure 7b). Additionally, the T2 relaxation time between CD4-SPION-labeled T cells at 24 h and 72 h post-infusion time point shows no significant difference (*p* = 0.637, Figure 7b). These findings demonstrate the feasibility of tracking helper T cells in vivo.

### 3.5. Histopathology Correlation

**Figure 8:**
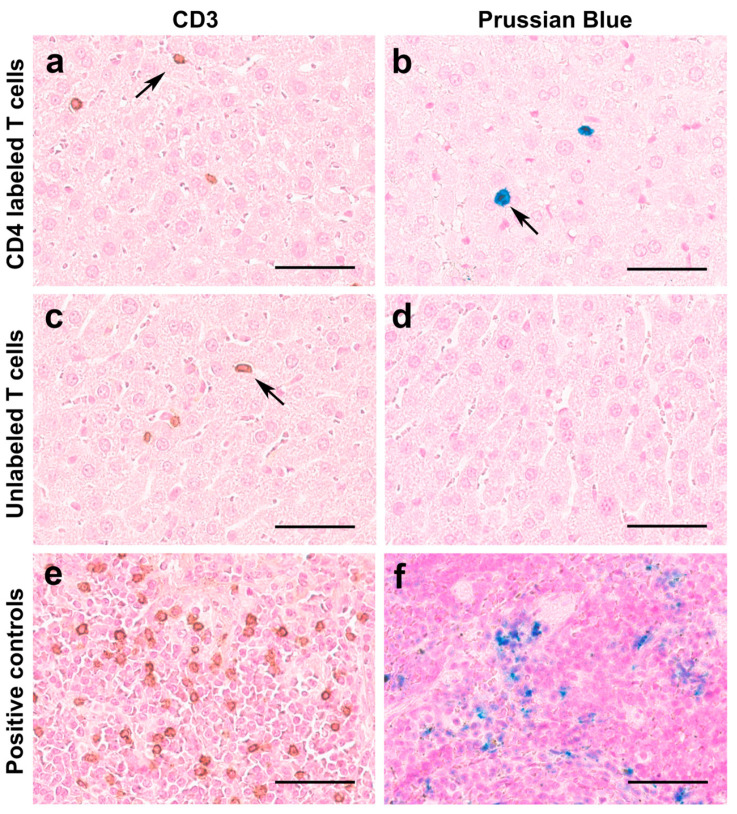
Histological analyses of T cell distribution in the liver and SPION retention. Representative microscopy images of liver tissues show prominent T cell infiltrates in both SPION-labeled T cell-receiving mice ((**a**), arrow) and unlabeled T cell-receiving mice ((**c**), arrow). Additionally, SPION-labeled T cells exhibit positive Prussian blue (iron) staining ((**b**), arrow), contrary to unlabeled T cells (**d**). Positive controls display human CD3 cells in brown (**e**) and iron deposits in spleen tissue (**f**). Scale bar represents 50 μm.

Histopathological staining of liver tissues confirmed the presence of numerous T cell infiltrates, as evident by the brown staining targeting human CD3 in both groups (Figure 8a,c). We did not see any difference in the number of T cells per high-power field in both groups. As expected, Prussian blue staining correlated with T cell immunohistochemistry findings (Figure 8b) in the labeled T cell group but not in the unlabeled T cell group (Figure 8d). These results confirm that the MRI signal decay measured results from CD4-SPION-labeled T cells that have homed to the liver.

## 4. Discussion

This study shows that CD4-SPIONs are effective T cell labeling contrast agents that can be used for subsequent MRI tracking. This is the first successful targeted labeling of CD4^+^ cells with a clinically relevant contrast agent. Indeed, CliniMACS CD4-SPION microbeads are currently approved for clinical research, and their CD34 counterpart is approved by the FDA as a Humanitarian Use Device. Off-label use of these SPION beads as a contrast agent would nonetheless require Investigational New Drug approval in future studies. All in all, this targeted labeling strategy is much more efficient than non-specific SPIO nanoparticles and may help bridge the gap in the detection limit of adoptive cell therapy on clinical scanners.

CD4-SPIONs were originally designed for magnetic cell sorting of CD4^+^ T cells [52], and the SPION is expected to eventually detach from the target after sorting. With our modified protocol, we have previously shown with CD25 [35] and now CD4, that the SPION can be compelled to internalize into the cells. We postulate that the specific antibody binding facilitates internalization of the SPION into CD4^+^ T cells by using clathrin-mediated endocytosis (CME), which naturally promotes internalization of T cell membrane proteins [32,48]. This process involves the formation of clathrin-coated pits on the plasma membrane. The pits collapse and squeeze to form vesicles, which transport internalized molecules into the cell [48,53] and later fuse with endosomes. Our understanding is that a cell needs to recycle its surface receptors frequently enough to enable full cargo endocytosis. This process may also increase in IL-2-activated T cells, leading to extensive endocytosis in the case of CD4^+^ cells [35]. This novel feature may prove instrumental in higher-plex sorting of multi-omics protocols [54] by isolating specific immune cell types using magnetic SPION beads, cleaving the bond on surface-associated SPION, and retaining only the cells with internalized SPION.

Numerous teams have attempted to label and track T cells using various magnetic particles. For example, Bulte et al. employed a biotin-streptavidin strategy, which resulted in cell surface tagging and showed limited in vivo tracking capability [55]. Jin et al. used Molday Ion to label mouse T cells in a mouse model of stroke and demonstrated the feasibility of using SPIOs for T cell tracking [45]. Our approach is cell-specific, which offers advantages beyond CD4^+^ cells, and enables maximal loading at much lower iron concentrations (2 vs. 12 μg Fe/mL). Also, our method has the advantage of not needing any additional manipulation of the cells, thereby minimizing possible cell functional changes and maximizing viability for improved safety in future clinical applications. Indeed, the CD4 SPIONs are already part of the standard workflow in the clinical isolation of the CD4^+^ T cells, hence minimizing the labeling and manipulation process.

While we have shown that CD4-SPIONs are effective in labeling the CD4^+^ T cells, they may also be internalized in phagocytic cells such as macrophages. Indeed, many macrophage tracking strategies rely on their non-specific uptake of large amounts of contrast agent, hence possibly introducing nonspecific signals in other models [35]. We have previously shown that the CD25^+^ T cells selectively uptake CD25-SPION while macrophages do not [35]. We believe the same holds true for the CD4-SPION; this becomes a major advantage over conventional contrast agents, thereby lessening confounding MRI signals [56]. The observed selectivity may be due to the fact that modification of the surface of the microspheres masks them from recognition and uptake by phagocytes. Although we confirmed preserved activation and cytokine production through standard flow cytometry, emerging high-throughput volumetric cytometry methods [57] may enable future assessment of spatial polarization or activation domains within living T cells, for rapid, quantitative assessments of internalization events with subcellular spatial resolution

The current standard of tagging and tracking T cells includes radionuclides for SPECT-CT and PET imaging. Some disadvantages of SPECT and PET include poor spatial resolution and challenges with prolonged cell tracking. Additionally, these techniques expose the cells to ionizing radiation and increase the risks of other adverse effects to the patient [58]. Here, we have demonstrated that CD4-SPION generates detectable MRI contrast in a 7T preclinical scanner. In principle, they could also be used successfully on low-field clinical MRI systems, with adequate adjustment of the pulse sequences applied [59]. This imaging-based cell tracking is non-invasive, non-radiative, and highly reproducible. Nonetheless, as for any proton-based contrast agent, including CD4-SPION, one can only assess cell infiltrates in a semi-quantitative manner and cannot provide absolute cell counts. Fluorine MRI could circumvent this limitation, but is overall less sensitive and not readily clinically translatable [24,60]. Magnetic particle imaging (MPI) is another quantitative method but suffers the similar limitations as fluorine MRI [61].

The capacity to non-invasively track CD4^+^ T cells in the broad adoptive cell therapy landscape can be a useful determinant of the success of such therapies [62,63]. In transplantation immunology, this could help decipher between graft rejection and the balance between effector and Treg cells [64,65,66,67]. Tracking homing and persistence is hereby an early indicator of treatment efficacy. This platform could also inform diabetes therapeutic strategies [68,69], as well as various autoimmune disease treatments [70,71]. More broadly, specifically targeting T cell subtypes and other immune cells at large could provide much-needed interplay and insights on how our immune system operates in health and disease.

To conclude, this study shows that CD4-SPIONs are an effective T cell labeling contrast agent that can be used for subsequent MRI tracking. This is the first instance of targeted labeling of CD4^+^ cells with a clinically relevant contrast agent. This safe, sensitive, and targeted approach may help overcome the detection limits of cell transplantation therapies using clinical MRI scanners in various disease conditions.

## Data Availability

The data presented in this study are available upon request.

## Appendix A

**Table A1:** Overview of T cell labeling strategies for MR imaging, detectability and limitations.

Contrast Agent	MR Detectability	Limitations
USPIO [42]	In vitro	No in vivo imaging or biodistribution tested
SPIO [72]	In vivo	Low specificity, Short tracking window
SPIO [45]	In vitro and in vivo	Not CD4 specific in vivo and relies on prior ex vivo labeling
USPIO [73]	In vivo	Limited spatial resolution, no quantification
SPIO-PLL [74]	In vitro & in vivo	Not CD4 specific
VSOP [75]	In vivo	Not CD4 specific, signal may reflect iron rather than live cells
FE-PLL [76]	In vivo	Not CD4 specific, limited sensitivity for lesions < 50 μm
MION [77]	In vitro	Not CD4 specific
